# Identifying Regional Key Eco-Space to Maintain Ecological Security Using GIS

**DOI:** 10.3390/ijerph110302550

**Published:** 2014-02-28

**Authors:** Hualin Xie, Guanrong Yao, Peng Wang

**Affiliations:** Institute of Poyang Lake Eco-Economics, Jiangxi University of Finance and Economics, Nanchang 330013, China; E-Mail: yaoguanrong@aliyun.com

**Keywords:** eco-space, ecological security, environmental sustainability, ecosystem services, GIS

## Abstract

Ecological security and environmental sustainability are the foundations of sustainable development. With the acceleration of urbanization, increasing human activities have promoted greater impacts on the eco-spaces that maintain ecological security. Regional key eco-space has become the primary need to maintain environmental sustainability and can offer society with continued ecosystem services. In this paper, considering the security of water resources, biodiversity conservation, disaster avoidance and protection and natural recreation, an integrated index of eco-space importance was established and a method for identifying key eco-space was created using GIS, with Lanzhou City, China as a case study. The results show that the area of core eco-space in the Lanzhou City is approximately 50,908.7 hm^2^, accounting for 40% of the region’s total area. These areas mainly consist of geological hazard protection zones and the core zones of regional river systems, wetlands, nature reserves, forest parks and scenic spots. The results of this study provide some guidance for the management of ecological security, ecological restoration and environmental sustainability.

## 1. Introduction

Ecological security and environmental sustainability are the foundations of sustainable development. Land use/land cover change not only bring enormous changes to the surface structure of landscapes, but also affect the flow of materials and energy through a region, leading to a profound impact on ecological security in cities [1,2,3,4,5]. In the past 50 years, urbanization, expansion of industrial land and construction of transport networks have caused an unprecedented destructive fragmentation of the natural environment and have been increasing the human footprint on natural ecosystems [2]. The loss of natural space caused by land use changes has become the main threat to ecological security [6]. Scholars have gradually realized that an explicit evaluation framework should be constructed to identify the importance of space sensitive to human and ecological connectivity. The evaluation and identification of sensitivity space for human habitation and biological species and the establishment of priority conservation areas is beneficial not only to improve their conservation value, but also to minimize the likelihood of losing them. 

With the continued expansion of urban and industrial land in China, a new land use type has appeared with importance for cities, agriculture, forestry, and environmental planning and government approvals. The new land use type, called ecological space (eco-space) [7], is different from the urban and rural space concept and includes nature reserves, drinking water source areas, country parks, wetland parks and ecologically important forests. Eco-spaces can be considered as the “liver” of a city, as they are important for ecosystem detoxification and the maintenance of ecological security. In recent years, land use changes induced by urbanization and industrialization have posed an enormous threat to eco-spaces including wetlands and woodlands. Eco-spaces in China are prominently threatened by two processes. First, urban expansion has turned many wetlands, woodlands and other eco-spaces, which play vital ecological roles, into built up areas, which have a great impact on ecological security and regional ecosystems. Second, due to the national arable land policy, wetlands and other eco-spaces are facing the threat of exploitation. The over-exploitation of eco-spaces will have disastrous consequences, including biodiversity loss, ecological degradation, and the decline of ecosystems’ regulating abilities.

A new comprehensive national land use plan (2006–2020) has been approved by the State Council in China. It has clearly outlined the requirements for eco-space, which are “based on the requirements of building an environmentally friendly society, which focus on building healthy living environments, and overall arrangements for living and protecting productive land give priority to the protection of natural eco-spaces, to promote the development of an ecological civilization”. This means that a project must specify the strict protection of key ecological land and the building of a strong ecological pattern. Key eco-spaces are an essential part of the nationally ecological security and sustainable economic and social development. Therefore, identifying key eco-spaces is an urgent need.

Key eco-space (or basic eco-space) is the space required to maintain the integrity and the continuity of a regional landscape pattern, to protect regional water resources, to protect biodiversity, to prevent geological disasters, and to conserve soil and water. Its key mission is maintaining the security and health of the land important for human life, and it can offers society with continued ecosystem services. It is the basic protection by which regional land ecosystems continue to provide ecosystem services. Therefore, identifying key regional eco-space has a vital role in maintaining ecological security and environmental sustainability. We can then develop a realistic strategy to manage the eco-space and carry out ecological restoration and construction.

Currently, identifications of key regional eco-space have primarily employed a single characteristic, e.g., water security, biodiversity conservation, soil erosion protection and green space [6,8,9,10,11,12]. For biodiversity conservation, Rouget *et al.* [8] developed a method to identify the spatial components of the ecological and evolutionary processes important for regional conservation planning using GIS. Vimal *et al.* [6] developed a multi-criteria assessment method to identify the vulnerable areas by three factors: The presence of rare or remarkable species, extensive areas of high ecological integrity, and landscape diversity. Zhang *et al.* [13] combined an ecological conceptual model of landscape with assessment methods to evaluate of ecosystem service functions and established a spatial analysis model for urban minimum ecological land using GIS. Moilanen *et al.* [10] developed a conservation assessment method to identify new priority areas that best meet desired the targets in combination with any existing PAS. This method, however, is likely to produce portfolios with a large number of small and isolated PAS, and such portfolios are less ecologically and economically feasible [14,15,16]. For identifying water security areas, Vos *et al.* [17] planned adaptation areas for wetland ecosystem to respond to the impact of climate change through the identification of key water security areas. By identifying the key water security areas, Brouwer and van Ek [9] analyzed the ecological, social and economic impacts of their protection and restoration. In addition, using GIS and the Universal Soil Loss Equation (USLE), Zagasa [18] identified the key areas for soil erosion protection on Mount Olympus, Greece.

The primary methods for identifying eco-space include the CA model, niche theory, graph theory and ecological network analysis. In addition, some scholars in China use the carbon-oxygen balance method and landscape security patterns to estimate the demand for eco-space [13,19]. However, there is little research on whether the identification of key eco-space maintaining the ecological security and ecological security. 

Based on the ecological suitability of land, natural ecosystem services, the continuity of natural landforms, existing land use patterns and the demand for ecological protection, it is necessary to promote the integrity and continuity of eco-space. In this study, we propose a suitable method to identify key eco-space using GIS. We seek to provide a foundation for ecological protection based on the land carrying capacity and to provide a reference method for the effective management of ecosystem health.

## 2. Identifying Framework

**Figure 1:**
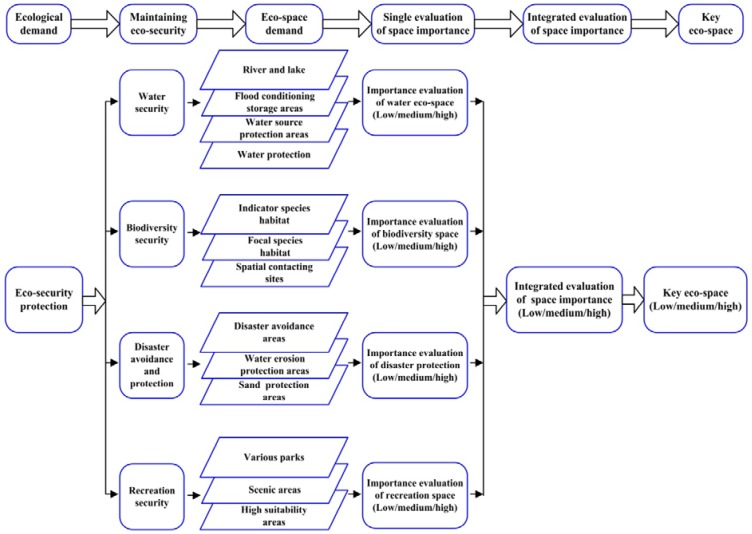
The framework for identifying key eco-space for ecological security.

The purpose of key eco-space is to maintain ecological security. As shown by previous studies [6,8,9,11,17,19], ecological security includes the maintenance of water security, biodiversity protection security, disaster avoidance and recreational area protection. The frame for identifying key eco-space is shown in Figure 1. 

## 3. Materials and Methods

### 3.1. Study Area

**Figure 2:**
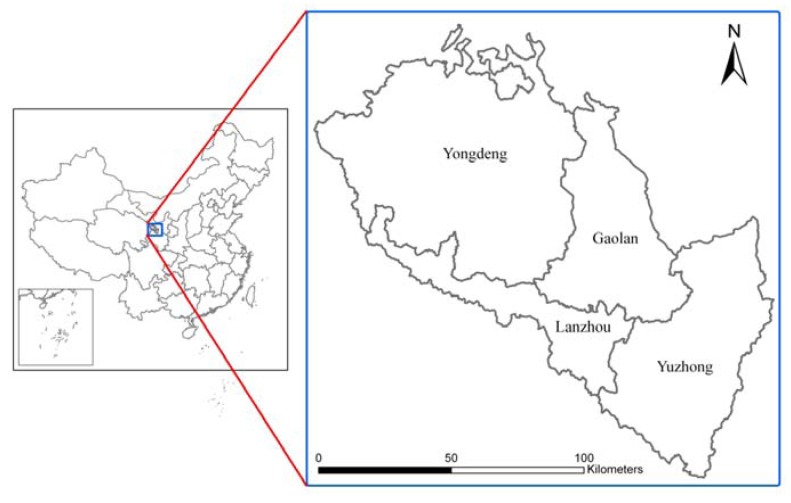
Study area.

Lanzhou City is located in the west of the Longxi Loess Plateau (see Figure 2), in a transition region between the Qinghai-Tibet Plateau and the Loess Plateau. Its northern edge is close to the Mongolian Plateau, and its western portion is an arid area of the Inland Northwest. Lanzhou City sits in a semi-arid climate zone, with an average annual temperature of 9.1 °C, an average annual precipitation of 324.8 mm and an average annual evaporation of 1,468 mm. Evaporation is approximately four times higher than precipitation, and drought is significant. The terrain is low in the northeast and high in the east and south. The Yellow River flows from southwest to northeast across the entire region. It contains three landform types, rocky mountain, loess hills and valley basin, accounting for 19%, 66% and 15% of the total area, respectively. Vegetation coverage is highly varied within the region. This factor reflects overall ecological characteristics and is a key factor in regional water conservation, soil conservation and ecological and environmental enhancement. The arid climate in the region has resulted in poor vegetation cover. In addition, human activities over the years have degraded the vegetation, thereby exacerbating soil erosion, desertification and the deterioration of the ecological environment. Lanzhou city’s administrative area comprises five districts and three counties: Yongdeng County, Yuzhong County, Gaolan County, Honggu District, Xigu District, Qilihe District, An’ning District and Chengguan District. In 2005, the study area had a total population of approximately 3.12 million, and the area of arable land was 2,713 hm^2^.

### 3.2. Data

This study uses the interpretation of remote sensing data and the spatial analysis of existing land use layers via Geographic Information Systems. The software platforms used are ArcGIS9.2 and ERDAS9.1. In this study, we used land use data and a DEM of 1:10,000 derived from the Land Surveying and Planning Institute of Lanzhou city. A soil map; a zoning map of Drinking Water Conservation Areas; a sensitivity analysis of landslide and debris flow; maps of nature reserves, forest parks, geological parks and scenic areas; and the basic geographic data of Lanzhou were obtained from the Cold and Arid Region Environmental and Engineering Research Institute, Chinese Academy of Sciences.

Before the analysis, we transformed all graphical data to the ALBERS equal-area conic projection (Krasovsky_1940_Albers). Through resampling, the spatial resolution was converted to a raster of 50 × 50 m. The two operations were conducted by projecting and resampling, respectively, to ensure that the projection and precision of the graphical output remained uniform.

### 3.3. Methods

The organization of this paper is as follows: first, we establish the eco-space importance indexes for maintaining water security, biodiversity, disaster protection and recreation. Second, we evaluate each indicator and assign a value to each ranking, and completing the single-factor importance index of eco-space. Final, we overlay the importance indexes of each single factor and complete the integrated identification index for regional key eco-space.

#### 3.3.1. The Framework for the Importance Evaluation of Eco-Space

Based on our evaluation objectives for the importance of ecological space and the existing methods for sensitivity analysis of different ecosystems, a three-level index system was established to evaluate the importance of ecological space [6,10,17,18]. In contrast to previous research [6,9,15,16], the three-level system accounts for various aspects of ecological security, including water security, biodiversity conservation, disaster avoidance/protection and recreation. The first level of the index is the object layer, namely, the integrated index of ecological space importance. The second level is the item layer, namely, the influencing factors for ecological importance, which include water security, biodiversity conservation, disaster avoidance and protection, and natural recreation. The third level is the index layer, which is the individual indicators measuring the influencing factors. The indicators are shown in Table 1.

**Table 1:** Evaluation index system for ecological space importance.

Object Layer	Item Layer	Index Layer
Integrated index of ecological space importance	Importance of maintaining water security	Distance index to a river or lake (*s_1_*)
		Type index of flood conditioning storage areas (*s_2_*)
		Importance index for water conservation (*s_3_*)
		Type index of water source protection areas (*s_4_*)
	Importance of biodiversity conservation	Sensitivity index of habitats (*s_5_*)
	Importance of disaster avoidance and protection	Sensitivity index of geological hazards (*s_6_*)
		Importance index of soil conservation(*s_7_*)
		Importance index of land desertification protection(*s_8_*)
	Importance of natural recreation	Suitability index of natural recreation(*s_9_*)

(1) Importance of maintaining water security

In this study, the first type of eco-space is that for water resource protection, water conservation and flood storage. Four indexes have been selected to evaluate the importance of this type of eco-space: the distance to a river or lake, the type of flood storage areas, the type of water source protection areas, and the importance for water conservation. Using the spatial distributions of rivers and lakes, the index of the *buffering distance to a river or lake* was obtained with the straight-line distance function in ArcGIS9.2. The index of the *type of flood conditioning storage areas* was obtained from maps of land use, wetlands and detention basins. The index of the *type of water source protection areas* was obtained from the digitized zoning map of water source protection areas. The index of the *importance index of water conservation* was obtained by differentiating between mountains, hills and plains and then overlaying these with the ecosystem types in these areas. The ranking of these eco-spaces’ importance for maintaining water conservation was obtained from the DEM and from ecosystem class data (see Table 2).

**Table 2:** Importance index for water conservation rankings.

Topographic Form	Ecosystem Class	Importance
Mountain	Forest or Wetland/ Steppe or Meadow / Desert	High / Relatively high / Medium
Hill	Forest or Wetland/ Steppe or Meadow / Desert	Relatively high / Medium / Relatively low
Plain	Forest or Wetland/ Steppe or Meadow / Desert	Medium / Relatively low / Low

Based on previous research [20], we developed the ranking standards for the four indexes, distance to a river or lake (s_1_), type of flood conditioning storage areas (s_2_), importance for water conservation (s_3_) and type of water source protection areas (s_4_), and we assigned values to each rank (see Table 2). 

We used a disjunction function to calculate the index of water security (ES_1_) for each grid, where the four indexes represent four aspects of maintaining regional water security. The Equation is as follows:

ES*_w_* = *Max* (*s*_1_, *s*_2_, *s*_3_, *s*_4_) (1)

where ES_w_ represents the importance index of an eco-space maintaining water security, s_1_ is the index of the distance to a river or lake, s_2_ is the index of the type of flood conditioning storage areas, s_3_ is the index of the importance for water conservation, and s_4_ is the index of the type of water source protection areas. 

Using Equation (1), we calculated the importance index of an eco-space maintaining water security for each grid Using ArcGIS9.2. Finally, using the raking standards in Table 2, we obtained a spatial map of water security importance.

(2) Importance of biodiversity protection

Maintaining the security of biodiversity requires identifying the key processes and spatial patterns of biodiversity conservation at the regional and landscape scale and developing an urban and rural continuous native habitats and biological corridor systems, thereby protecting the integrity and healthy of the regional ecosystem. In this study, we identified the key biodiversity eco-space with the sensitivity index of habitats (s_5_). Using the land use type and the service equivalent quantum of biodiversity established by previous studies, we calculated the biodiversity service values of woodlands, garden plots, arable lands and wetlands. The equivalency factor for the biodiversity of garden plots was calculated by averaging the values of woodlands and grasslands. Then, we corrected the service values with the protection rankings of different land use types. The Equation for the sensitivity index of habitats (s_5_) is as follows:

*S*_5_ = *n*_1_ × *m* (2)

where, s_5_ is the sensitivity index of habitats, *n*_l_ is the service equivalent quantum of biodiversity for land use type *l* and *m* is the corrected value of the protection ranking. Nature reserves, parks, scenic areas and other land use types were assigned values of to 1.75, 1.5, 1.25 and 1, respectively.

Using Equation (2), we used the spatial analysis module in ArcGIS9.2 to calculate the sensitivity index of habitats for each spatial unit. By using the standard deviation (1Std Dev) classification, the result of evaluate the sensitivity of a habitat was divided into five rankings (extremely sensitive, highly sensitive, moderately sensitive, mildly sensitive and not sensitive). 

(3) Importance of disaster avoidance and protection

Eco-space that maintains disaster avoidance and protection is made up primarily of zones protecting against geological hazards, soil erosion and land desertification. In this study, we selected three indexes types of geological hazard zones (s_6_), importance for soil conservation (s_7_) and importance for land desertification protection (s_8_), to obtain the importance index for disaster avoidance and protection. Geological hazard area types were obtained from sensitivity evaluation of the geological hazards in the study area including landslides and debris flows.

The soil conservation and land desertification protection importance indexes were obtained by overlaying the spatial distribution map of ecosystem types and the sensitivity evaluations of soil erosion and land desertification, using the ranking standards in Table 3. Using the universal soil loss equation (USLE), we evaluated the soil erosion sensitivity of the study area. The USLE includes five factors rainfall and runoff (R), soil erodibility (K), slope and slope length [20] and crop/vegetation and management (C). We evaluated land desertification sensitivity, using humidity index, the days with wind speed greater than 6 m/s in winter and spring, soil texture and vegetation coverage in winter and spring.

**Table 3:** Importance rankings for soil conservation and land desertification protection.

Eco-space Type	Degree of Sensitivity to Soil Erosion and Land Desertification	Importance for Soil Conservation and Land Desertification Protection
Forest ecosystem Steppe ecosystem Meadow ecosystem Desert ecosystem	Extremely sensitive	High
	Highly sensitive	Relatively high
	Moderately sensitive	Medium
	Mildly sensitive	Relatively low
	Not sensitive	Low

We used a disjunction operation to calculate the importance index of disaster avoidance and protection (ES_3_) for each grid. The three indexes of geological hazard sensitivity, importance for soil conservation and importance for land desertification protection reflect different ecological security issues. The Equation is as follows:

*ES_d_* = *Max* (*s*_6_, *s*_7_, *s*_8_) (3)

where ES_d_ represents the importance index of eco-spaces maintaining disaster avoidance and protection, s_6_ is the sensitivity to geological hazards, s_7_ is the importance for soil conservation, and s_8_ is the importance for land desertification protection. 

(4) Importance of natural recreation 

Residents enjoy the natural environment through tourism and active involvement in recreational activities. For public recreational activities, natural landscapes are more superior to other landscapes. In this study, eco-space for recreation security refers to natural landscape elements and space, giving key significance to the quality of the recreational experience. Therefore, eco-space for recreation security consists of existing scenic spots, forest parks, geological parks, and ecological space with high potential for recreation. In this study, we identified natural recreation land with a natural recreation suitability index (s_9_). In general, ecosystems with high entertainment value may be a place for residents to enjoy natural recreation activities. Based on land use type and the service equivalent quantum of entertainment/culture established by previous research, we corrected the service equivalent quantum for recreation. The recreational equivalency factor for garden plots was calculated by averaging the values of woodlands and grasslands, while others were held constant. We calculated the recreation service values of woodlands, orchards, arable lands and wetlands, and then corrected the service value using the recreation ranking. The formula for the natural recreation suitability index (s_9_) is given as follow:

*s*_9_ = *p_l_* × *q* (4)

where, s_9_ is the index of natural recreation suitability, *p_l_* is the service equivalent quantum for entertainment/culture for land use type *l* and *q* is the corrected recreation ranking value. Parks, scenic areas, nature reserves and other lands were assigned the values of 1.75, 1.5, 1.25 and 1, respectively.

Using the Equation (4), we conducted spatial analysis in ArcGIS9.2 to calculate the natural recreation suitability index for each spatial unit. Using the standard deviation (1Std Dev) classification, natural recreation suitability was divided into five rankings (extremely suitable, highly suitable, moderately suitable, less suitable and least low suitable). 

#### 3.3.2. Criteria for the Indexes of Eco-Space Importance Evaluation

For the different security factors, impacts and contributions, each indicator has varying degrees. We use consulted experts to assign scores to the ranks of each single factor. Twenty-five experts in ecology, geography, land science, urban and rural planning and water resources were consulted by email. We received feedback from 20 experts and averaged their responses to determine the final score for each rank. Each rank, extremely important, moderately important, generally important or not important, was assigned values of 4, 3, 2 and 1, respectively. The ranking criteria and assigned scores for the indexes of eco-space importance evaluation are listed in Table 4.

**Table 4:** Ranking criteria and assigned scores for the indexes of eco-space importance evaluation.

Index	Extremely Important	Moderately Important	Generally Important	Not Important
Distance index to a river or lake (*s_1_*)	<50 m	50~100 m	100~150 m	>150 m
Type index of flood conditioning storage areas (*s_2_*)	Wetland areas	Core areas of detention basins	Non-core areas of detention basins	Other areas
Importance index for water conservation (*s_3_*)	High	Relatively high	Medium	Relatively low/ Low
Type index of water source protection areas (*s_4_*)	First-grade protection zones of water resources	Second-grade protection zones of water resources	Quasi-watershed protection zone	None
Sensitivity index of habitats (*s_5_*)	Extremely sensitive	Highly sensitive	Moderately sensitive	Mildly sensitive/ Not sensitive
Sensitivity index of geological hazards (*s_6_*)	First-grade protection zones of water resources	Second-grade protection zones of water resources	Quasi-watershed protection zone	None
Importance index for soil conservation (*s_7_*)	Extremely sensitive	Highly sensitive	Moderately sensitive	Mildly sensitive/ Insensitive
Importance index for land desertification protection (*s_8_*)	Extremely sensitive	Highly sensitive	Moderately sensitive	Mildly sensitive/ Not sensitive
Suitability index of natural recreation (*s_9_*)	High	Relatively high	Medium	Relatively low/ Low
Assigned score	4	3	2	1

#### 3.3.3. Integrated Evaluation of Eco-Space Importance

The importance of an eco-space derived from a single-factor analysis only reflects the effect of each factor. To reflect the integrated differences in the importance of regional eco-spaces, we need to use the assigned values for every ranking of the above factors and calculate an integrated index of eco-space importance. Its equation is as follows:

*ES* = *Max* (*ES_w_*, *ES_b_*, *ES_d_*, *ES_r_*) (5)

where, *ES* is the integrated index of eco-space importance, *ES_w_* is the importance index of eco-space maintaining water security, *ES_b_* is the importance index of eco-space maintaining biodiversity conservation, *ES_d_* is the importance index of eco-space maintaining disaster avoidance and protection, and *ES_r_* is the importance index of eco-space maintaining natural recreation.

Using Equation (5), we calculated the comprehensive eco-space index for each grid using ArcGIS9.2. This resulted in a spatial plot of eco-space importance.

#### 3.3.4. Identification of Key Eco-Space

**Figure 3:**
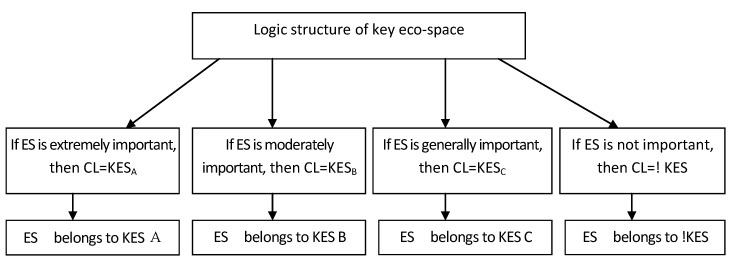
Model for identifying key eco-space.

To identify the key eco-space, it was necessary to systematically determine the spatial structure of the regional ecosystem. The purpose of this was to provide a possible spatial strategy for regional development and, at the same time, to limit the exploitation of the core area of ecological conservation and to optimize the use of eco-space. Based on the integrated evaluation of eco-space importance, the model for identifying key eco-space is presented in Figure 3. 

Setting CL as the indicator variable for eco-space, there are four possible states: extremely important, moderately important, generally important and not important (given the values 4, 3, 2 and 1, respectively). Thus, we define four types of key eco-space. These types are core eco-space (KES_A_, type A), which is the primary eco-space for maintaining ecological security, assistive eco-space (KES_B_, type B), transitional eco-space (KES_C_, type C), and non-key eco-space (!KES). The structure and pattern of a KES can be identified through the processing model in Figure 3. The results provide a database to inform strategies for regulating regional eco-space development.

## 4. Results and Discussion

Using the above evaluation indexes and methods of identifying ecological space, we obtained the results of the single-factor and integrated evaluations of the eco-space importance in Lanzhou city using ArcGIS9.2 (see Table 5). 

**Table 5:** Results of the importance evaluations of eco-space in Lanzhou City.

Evaluation Factors	Importance Ranking	Areas (hm^2^)	Percentage of Total Area (%)	Cumulative Percentage of Total Area (%)
Water security	Extremely important	6,764.375	5.22	5.22
	Moderately important	11,156.1	8.61	13.83
	Generally important	34,970.05	26.99	40.82
	Not important	76,693.825	59.18	100
Biodiversity conservation	Extremely important	5,810.65	4.48	4.48
	Moderately important	5,222.725	4.03	8.51
	Generally important	4,441.3	3.43	11.94
	Not important	114,109.675	88.06	100
Disaster avoidance and protection	Extremely important	46,461.4	35.85	35.85
	Moderately important	37,533.375	28.96	64.81
	Generally important	6,194.7	4.79	69.60
	Not important	39,394.875	30.40	100
Natural recreation	Extremely important	4,688.425	3.62	3.62
	Moderately important	6,289.425	4.85	8.47
	Generally important	2,435.725	1.88	10.35
	Not important	116,170.775	89.65	100

**Figure 4:**
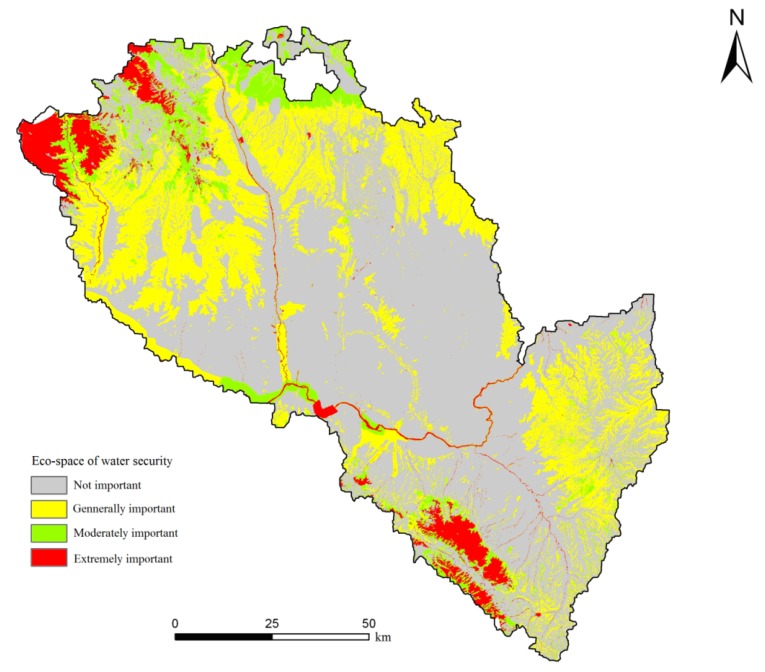
Evaluation results of the importance of water security eco-space in Lanzhou City.

As seen in Figure 4 and Table 5, the areas of extremely important and moderately important water security eco-space are 6,764.375 hm^2^ and 11,156.1 hm^2^, respectively, together accounting for 13.83% of the total area. These areas are extremely valuable for the maintenance of local water security, including water conservation and flood regulation and storage. Figure 4 indicates that the eco-spaces important for water security are mainly woodlands and regional river systems, which located in the northwest and southern mountains. This is primarily because the woodlands in these areas have good quality and significant water conservation functions. Meanwhile, the lake-river system in these areas plays a vital role in flood control. These areas are the region's main sites for water conservation and flood regulation and storage and where should be strictly protected from exploitation.

**Figure 5:**
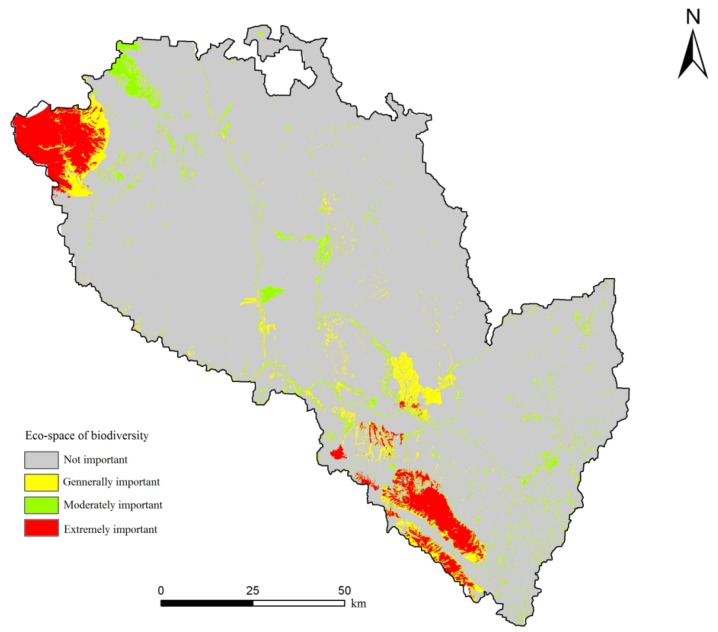
Evaluation results of the importance of biodiversity conservation eco-space in Lanzhou City.

The results for biodiversity conservation importance in Lanzhou City are shown in Figure 5. From Figure 5 and Table 5, the area extremely important for maintaining biodiversity is 5,810.65 hm^2^, accounting for 4.48% of the total area. These areas consist primarily of the core areas of Xinglong Mountain Nature Reserve and Tulugou National Forest Park. The sunny and half-sunny slopes of these areas contain deciduous broad-leaved forest, consisting mainly of aspen, birch and Liaodong oak, which is the core habitats for many species and should be strictly protected. The area of moderate importance for maintaining biodiversity is 5,222.725 hm^2^, accounting for 4.03% of the total area. This area consists mainly of woodlands located in the northwest mountains, which act as a buffer for native species. The areas of extremely important, moderately important and generally important for maintaining biodiversity make up close to 11.94% of the total area. Woodlands in the northwest and the south of the study area may be large habitat patches for maintaining biodiversity. Although the key areas for biodiversity conservation are small, they cover most of the habitats of regionally rare species and key species. Therefore, the key for maintaining regional biodiversity security is to identify the processes and spatial patterns of biodiversity conservation and to develop an urban and rural local continuous habitation and bio-corridor system. We should employ strict measures and dynamic monitoring in these areas and make considerable efforts in forestation, returning farmland to forest and grassland, and avoiding development activities in these areas.

**Figure 6:**
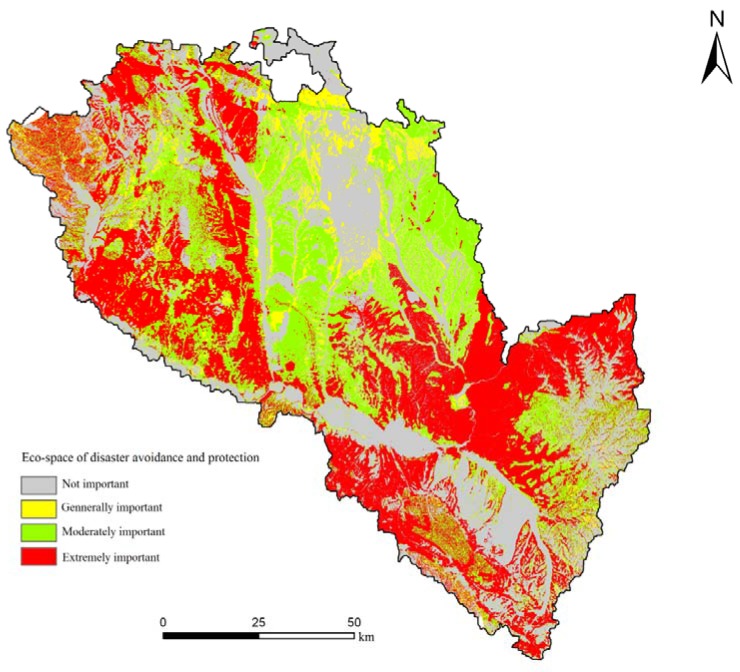
Evaluation results of disaster avoidance and protection importance in Lanzhou City.

As seen in Figure 6 and Table 5, for disaster avoidance and protection, the area of extreme importance is 46,461.4 hm^2^, accounting for 35.85% of the total area. These areas are extremely dangerous as geological hazards such as landslide and mudflows occur frequently. For the security of residents, development of land in these areas should be avoided. Extremely dangerous areas account for more than 30% of the total area, which means that the ecological environment is poor in most regions of Lanzhou City. There exist are serious hidden dangers from landslides, mudslides and other geological disasters in the Lanzhou Basin because of the spiking upward slope and the concentrated rainfall. At the same time, the region is characterized by sparse vegetation, thick loess cover, and severe soil erosion. Large areas prone to geomorphic hazards, such as landslides, mudslides and soil erosion, should be regarded as the emphasis region of evasion and protection. The area of moderate importance is 6,194.7 hm^2^, accounting for 28.96% of the total area. Most of these areas have a slope greater than 25 degrees, and the ground is barren and extremely sensitive to soil erosion and land desertification, and they make up the fundamental protection area for soil erosion. 

**Figure 7:**
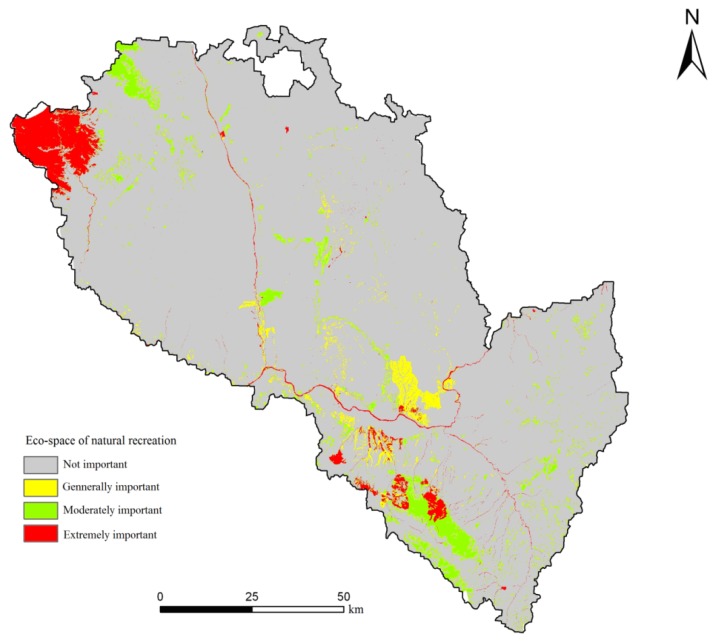
Evaluation results of natural recreation importance in Lanzhou City.

From Figure 7 and Table 5, for natural recreation importance, the area of extreme importance is 4,688.425 hm^2^, accounting for 3.62% of the total area. These areas are mainly located in the mountains and the northwestern river systems and are made up primarily of the core areas of forest parks, such as the forest parks of Tulugou, Xujiashan, Shifogou and Lanshan, and scenic spots, such as the scenic spots of Zhufushan and Guantangou. The primary in these areas land use types are wetlands and woodlands, which have high values for natural recreation. This is mainly because, with the growing demand for outdoor recreation, wetlands and forests have become the residents’ recreational open space. The area of moderate importance is 6,289.425 hm^2^, accounting for 4.85% of the total area. The recreational value of these areas is quite high and they are distributed in the northwest mountains and the non-core area of the Xinglong Mountain Nature Reserve. We ought to control their capacity for tourists, avoid overexploitation from tourism, and maintain the natural landscape with high recreational value in these areas.

**Figure 8:**
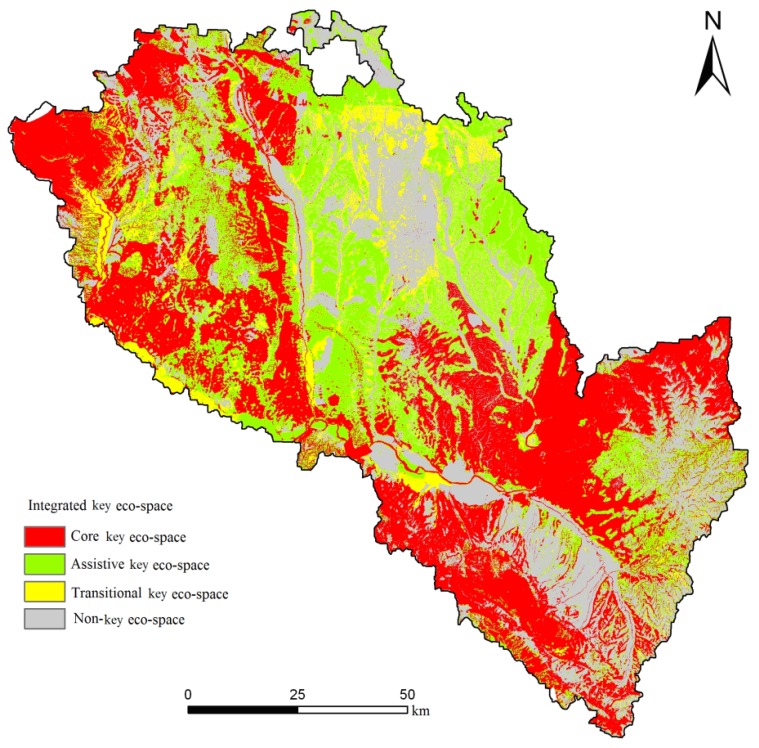
Spatial distribution of integrated key eco-space in Lanzhou City.

Using the model for identifying regionally key eco-space, we obtained the integrated key eco-space (see Table 6 and Figure 8) in Lanzhou City. 

From Figure 8, we see that the core eco-space consists of mountains and is mainly located in the northwest, east and south. These areas are made up primarily of the geological hazards of protection zones and the core areas of the regional river systems, wetlands, nature reserves, forest parks and scenic spots, which make up the ecological barrier to maintain ecosystem security. 

**Table 6:** Key eco-space types in Lanzhou City.

Type of Key Eco-space	Areas (hm^2^)	Percentage of Total Area (%)	Cumulative Percentage of Total Area (%)
Core key eco-space (Baseline security level)	50,908.7	39.29	39.29
Assistive key eco-space (Medium security level)	39,004.95	30.10	69.39
Transitional key eco-space (Ideal security level)	6,896.425	5.32	74.71
Non-key eco-space	32,774.275	25.29	100

The area of core eco-space is 50,908.7 hm^2^, accounting for 39.29% of the total area. This area is the bottom line of ecological land for the maintenance of regional eco-security and should be strictly protected and included in prohibited exploitation zones, and prohibiting any exploitation and construction activities. The area of assistive ecological land is 39,004.95 hm^2^, accounting for 30% of the total area. These areas are distributed in the northern and central regions. Core and assistive key eco-space together make up close to 70% of the total area. These areas are the key eco-spaces for maintaining water and soil ecological security and biodiversity, and should be heavily protected against the development and construction. The area of non-key eco-space is 32,774.275 hm^2^, accounting for 25.29% of the total area, and it mainly consists of built-up areas and arable land for human habitation and agricultural production.

Many cities (such as London, Frye Lane, Canberra, Paris and Vancouver) attach great importance to the protection of ecologically important land and maintain the proportion of ecologically important land at more than 50% of their total area. The proportion of core eco-space is close to 40% of the total area, which is similar to cities in foreign countries. The proportion of key eco-space (core, assistive and transitional key eco-space) in Lanzhou City is close to 75%. The identification of key eco-space in this article fully reflects the characteristics of the environment in the study area. The large proportion of key eco-space in Lanzhou City is primarily due to the large areas prone to land desertification and geomorphic hazards. For the areas of assistive and transitional key eco-space, it should reasonably be planned, developed and strictly control the scale.

Multi-criteria overlays to apportion spaces into different use values can be very valuable in identifying land use conflicts and prioritizing areas for protection or development, but they can also obscure pertinent data in the original layers. To reflect the integrated differences in the importance of regional eco-spaces, the smallest limiting factor method was used to calculate an integrated index of eco-space importance in this study.

In addition, we should consider some socio-economic factors in identifying regional key eco-space, for example, urban development, population growth and arable land protection policies. Programs for urban development and policies for arable land protection will affect key eco-space.

## 5. Conclusions

As shown by previous studies [6,8,9,11,17,19], the method for identifying key eco-space in this study improves on the existing ones in several ways. First, the method put forward in this paper is based on the need for ecological security. Second, compared to previous methods, it considers a wide range of factors for ecological security, including water security, biodiversity conservation, disaster avoidance and protection, and natural recreation. Third, using GIS and the scenario analysis method, it proposes spatially explicit and feasible multi-scenarios, which facilitate the effective management of ecological space.

Our results reveal the spatial characteristics of the key eco-spaces that maintain the security of water, biodiversity, disaster protection and recreation, indicating that this method of identifying key eco-space is feasible.

The area of core eco-space accounts for 40% of the total area of the study site and includes the protection zones for geological hazards and the core areas of regional river systems, wetlands, nature reserves, forest parks and scenic spots. These eco-spaces are the bottom line for maintaining ecological security [19].

With the acceleration of urbanization in China, increasing human activities will generate greater impacts on the eco-spaces that maintain ecological security. Therefore, it is extremely beneficial to explore key eco-space to provide the guidance for ecological management, ecological conservation and sustainable development.
